# 100 Jahre Facharzt für Urologie: Zur Ausbildung eines medizinischen Faches im Kontext von Spezialisierung und Professionalisierung

**DOI:** 10.1007/s00120-024-02409-0

**Published:** 2024-08-13

**Authors:** Friedrich H. Moll, Matthis Krischel

**Affiliations:** 1 Institut für Geschichte, Theorie und Ethik der Medizin, Centre for Health and Society, Heinrich-Heine-Universität Düsseldorf, Düsseldorf, Deutschland; 2 Deutsche Gesellschaft für Urologie, Düsseldorf, Berlin, Deutschland; 3 Urologische Klinik, Urologischer Arbeitsplatz, Krankenhaus Merheim, Kliniken der Stadt Köln gGmbH, Neufelder Straße 32, 51067 Köln, Deutschland

**Keywords:** Facharztausbildung, Bremer Richtlinien von 1924, Spezialisierung, Professionalisierung, Medizingeschichte, Medical specialty training, Bremen guidelines of 1924, Specialization, Professionalization, History of medicine

## Abstract

Dieser Artikel untersucht die Entwicklung der Urologie als eigenständige medizinische Disziplin in Deutschland mit besonderem Fokus auf die Professionalisierung und Spezialisierung im 19. und 20. Jahrhundert. Basierend auf historischen Quellen beleuchtet der Text die Bedeutung der Weiterbildungsordnungen der deutschen Ärzteschaft als Instrument der ärztlichen Selbstverwaltung und die Einordnung der Urologie als Sonderfach der Medizin in den Bremer Richtlinien von 1924, welche den Facharzt für Erkrankungen der Harnorgane (Urologie) etablierten.

## Abstract (en)

This article examines the development of urology as an independent medical discipline in Germany, with a particular focus on professionalization and specialization in the 19th and 20th centuries. Based on historical sources, the text illuminates the importance of the German medical profession’s further training regulations as an instrument of medical self-administration and the classification of urology as a medical specialty in the Bremen guidelines of 1924, which established board certification in diseases of the urinary organs (urology).

## Einleitung

Der ehemalige Präsident der Bundesärztekammer Hans Dietrich Hoppe (1940–2011) bemerkte 1997 in einer Publikation zum 100-jährigen Jubiläum der Institution:

„Neben der Berufsordnung (einer Art Disziplinarordnung) der deutschen Ärzteschaft ist die Weiterbildungsordnung (früher: Facharztordnung) das sowohl für Ärztinnen und Ärzte, aber auch für die gesamte Bevölkerung bedeutendste Instrument der ärztlichen Selbstverwaltung“ [1]

Und schon 1922 definierte ein Zeitzeuge den Status eines medizinischen Sonderfaches:

„Ein Sonderfach muß als ein in sich abgeschlossenes Gebiet der Heilkunde oder als Gruppe von zusammengehörigen derartigen Gebieten bereits ungeteilte Anerkennung genießen und muß zu seiner Ausübung eine besondere wissenschaftliche oder technische Vorbildung voraussetzen, wie sie das medizinische Universitätsstudium und das praktische Jahr allein nicht zu bieten vermag“ [2].

Diese Zitate umreißen die Aspekte von Professionalisierung und Spezialisierung im Bereich der Medizin im Allgemeinen und der Urologie im Speziellen. Eine zentrale Rolle für Deutschland spielen dabei die Leitsätze zur Facharztfrage von 1924, in der Literatur auch als Bremer Richtlinien bezeichnet, in denen die „Erkrankungen der Harnorgane (Urologie)“ als ein Sonderfach der Medizin genannt werden, für das Fachärzte bezeichnet werden sollten [3].

## Disziplinenbildung, Professionalisierung und Spezialisierung

Die akademische Disziplinenbildung ist ein Vorgang funktionaler Differenzierung, an dem das Wachstum des Wissens, der Forschungs- und Lehrstätten sowie Erfordernisse der Arbeitsteilung ursächlich beteiligt sind. Die akademische Fächerstruktur entstand in eigendynamischen Wachstumsprozessen, die ganz wesentlich von dem Autonomiestreben der beteiligten Akteure geleitet waren [4]. Die Entstehung und Entwicklung neuer Disziplinen folgte nur selten dem von dem Wissenschaftsphilosophen Thomas Kuhn (1922–1996) beschriebenen Muster revolutionärer Umbrüche im Wissenschaftssystem [5], sondern vielmehr einem evolutionären Modell der allmählichen Ausdifferenzierung und Verselbständigung neuer Forschungsrichtungen [6, 7].

Auf analytischer Ebene sind hier Professionalisierung und Spezialisierung wichtige Einordnungskategorien. Hierbei meint Professionalisierung den Prozess der Entwicklung einer Berufsgruppe mit einer gewissen Autonomie in der Leistungskontrolle. In einem weiten Sinn bedeutet Professionalisierung den Übergang von Tätigkeiten zu bezahlter Arbeit, die gewissen einklagbaren Qualitätsstandards unterliegt. In diesem weiten Sinne können Personen und Tätigkeiten sich professionalisieren – sie gewinnen an Professionalität [8]. Auf der kollektiven Ebene ist mit Professionalisierung die fachliche Entwicklung und Profilierung sowie die (potenzielle) Akademisierung von ganzen Berufsgruppen sowie Arbeits- und Handlungsfeldern gemeint, die mit der Etablierung von Professionen verbunden sein kann [9–11]. Das Untersuchungsfeld schließt für die Medizingeschichte auch immer einen Fokus auf sich wandelnde Arzt-Patienten-Verhältnisse, die Auseinandersetzung mit Laienbehandlern, der Rolle des medizinischen Erkenntnisfortschritts und der Ausdehnung des Krankenhauswesens mit ein [12, 13]. Der ärztliche Berufsstand gilt neben den Juristen und Theologen als voll professionalisiert [14, 15]. Für professionalisierte Berufsstände gibt es besondere Kontrollen der Zugänge zum Beruf und besondere Einwirkungen des Staates auf den Beruf. Die Professionen können aber selber ihre Marktlage beeinflussen, um ein Monopol zu erlangen und um sich Dominanz in der Arbeitsteilung und Autonomie in der Gestaltung ihrer Berufstätigkeit zu schaffen [16, 17].

Das Phänomen der Spezialisierung innerhalb der medizinischen Wissenschaften wird über die Urologie hinaus grundsätzlich international untersucht. Einige Autoren rekurrieren auf historische Referenzpunkte, um einen quasi zwangsläufigen, naturgesetzlichen, deterministischen Charakter der Spezialisierung zu postulieren [18–22]. Zumindest retrospektiv lassen sich Hochphasen der medizinischen Spezialisierung rekonstruieren [23]. Hier ist es auch einleuchtend, wenn die Differenzierung von Fächern in der Medizin und den Wissenschaften als Zeichen für die Spezialisierung einer Disziplin herangezogen wird. Während im Jahre 1910 im Durchschnitt an jeder deutschen medizinischen Fakultät 17 Einzelfächer existierten, lag die Zahl im Jahre 1850 noch bei ungefähr 5 [24].

Tatsächlich handelt sich bei der Spezialisierung innerhalb der Medizin (vgl. Tab. 1) natürlich nicht um eine deterministisch wirkende, mystische Kraft, welche die Entwicklung im 19. und 20. Jahrhundert bestimmte und bis heute bestimmt, sondern um den Versuch einer Beschreibung der Ausdifferenzierung von Arbeitsfeldern. Diese Ausdifferenzierung und die dabei verfolgten Interessenlagen wirken wiederum zurück auf die beteiligten Akteure. Sie prägten und prägen deren Selbstverständnis und Habitus. Das, was als Spezialisierung begriffen wird, wirkt dabei trotz aller Möglichkeiten, Stadien zu differenzieren, weniger gerichtet als ergebnisoffen. Der Prozess muss als historisch kontingent verstanden werden [25–28].

**Tab. 1:** Merkmale einer selbstständigen medizinischen Fachdisziplin. (Nach Eulner, Guntrau und Laitko [29, 30])

Eigene Geschichte
Eigener Name
Abgegrenztes Organsystem
Facharztanerkennung
Eigene Kliniken
Eigenständige Vertretungen an den Universitäten
Fachspezifisches Instrumentarium und eigenständige Behandlungsmethoden
Eigene wissenschaftliche Publikationsorgane
Eigene wissenschaftliche und berufspolitische Organisationen und Kultur

## Forschungsstand

Übersichten zur Etablierung klinischer Spezialfächer bieten George Rosen, [31] Hans Eulner [32] und George Weisz [33–36]. Speziell für die Chirurgie sind hier Ira Rutkov [37] und Thomas Schlich [38, 39] zu nennen. Häufig wird auf die Etablierung von Universitätskliniken und Krankenhausabteilungen mit entsprechenden Ausbildungsmöglichkeiten in unterschiedlichen Fragestellungen oder auf die Etablierung von Fachgesellschaften oder Fachzeitschriften in der Literatur abgehoben [40]. Schon Fritz Schultze-Seemann (1916–1987), zweiter Archivar der Deutschen Gesellschaft für Urologie e. V. (DGU), widmete in seiner Darstellung der Geschichte der DGU der Zeitschriftenentwicklung als konstituierendem Merkmal für eine Fachgesellschaft dem Thema einen breiteren Raum [41].

**Abb. 1:**
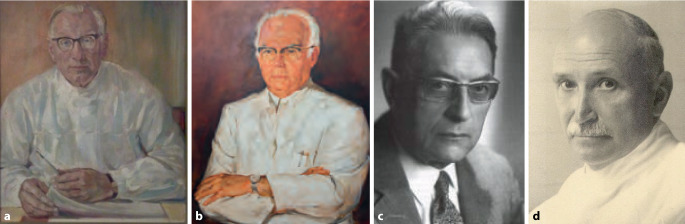
**a** Martin Stolze (1900–1989), Ölbild, Privatbesitz – Dr. K J. Stolze, Ulrich Bewersdorff (1920–2008), Universitätszeichenlehrer und freischaffender Maler in Halle (Repro Stolze, mit freundl. Genehmigung). **b** C.E. Alken (1909–1986; Universität des Saarlandes, mit freundl. Genehmigung). **c** Richard Übelhör (1901–1977) Urologie AKH Wien, Leitung als a o Professor 1963, Lehrstuhl für Urologie, Universität Wien 1967. **d** Hans Wildbolz, Leitung der nicht-klinischen chirurgischen Abteilung, Inselspital Berlin, o Professor ad personam! 1940 (Bildarchiv Dt. Gesellschaft für Urologie, Repro Moll-Keyn, mit freundl. Genehmigung)

Legte man die Etablierung von Lehrstühlen (ordentlichen Professuren) als Maßstab der Fachentwicklung an, so wäre von einer späten Etablierung der Urologie in Deutschland auszugehen. Im Anschluss an das erste Ordinariat für Urologie an der Universität Berlin im Jahr 1937 (Otto Ringleb [1875–1946]; [42, 43]) waren es v. a. Berufungen in der Nachkriegszeit: in Halle im Jahre 1958 (Martin Stolze, 1955 a o Professor, Oktober 1958 o Professor [44]); Berlin West 1963 (Wilhelm Brosig 1958 Extraordinarius, 1959 o. Professor mit Lehrstuhl); im in dieser Phase zu Frankreich gehörenden Saarland (Carl-Erich Alken 1909–1986, 1947 Habilitation Chirurgie (1); 1948 a o Professor Universität Nancy, 1958 o Professor Universität des Saarlandes).^1^ Außerhalb Deutschlands waren es im deutschsprachigen Bereich: in Österreich 1967 Richard Übelhör (1901–1977), a. o. Professor 1945, 1963 eigene Abteilung am AKH Wien, o. Professor 1967 [45], in der Schweiz Hans Wildbolz sen (1873–1940, 1919 a. o. Professor für urologische Diagnostik, 1940 o. Professor ad personam; [46]; vgl. Abb. 1).

Erste eigenständige urologische Abteilungen außerhalb der Universitätskliniken waren jedoch bereits deutlich früher etabliert (vgl. Tab. 2).

**Tab. 2:** Urologische Abteilungen im deutschen Sprachraum bis 1933. (Nach Halling [47])

Ort	Jahr	Krankenhaus	Gründer	Organisation
Wien	1872	Allg. Wiener Poliklinik	Robert Ultzmann (1842–1889)	„Sektion“ mit Betten
Wildungen	1904	Helenenheim	Wilhelm Schultheiss (1865–1935)	Chir. urol. Abteilung
Berlin	1906	St. Hedwig	Rudolf Jahr (1876–1965)	Belegabteilung bis 1922
Halle	1906	Klinik Weidenplan	Otto Kneise (1875–1953)	Privates Krankenhaus
Dortmund	1909	Barmherzige Brüder	Carl Schramm (1876–1949)	Belegabteilung
Bern	1912	Inselspital	Hans Wildbolz (1873–1940)	Hauptabteilung
Wildungen	1912	„Klinik Glückauf“	Hans Schultheiss (1865–1935)	Privatklinik
Hannover	1913	K H Siloah	Georg Prätorius (1878–1944)	Hauptabteilung
Düsseldorf	1925	Klinik Golzheim	Peter Janssen (1874–1947)	Privates Krankenhaus
Berlin	1927	Kaiserin-Auguste-Viktoria KH	Joachim Stutzin (1878–1954)	Hauptabteilung
Frankfurt a. M.	1930	Elisabeth KH	Hermann Schmutte (1896–1967)	Hauptabteilung
München	1932	Josephinum	Ludwig Kiehlleuthner (1876–1972)	Privates Krankenhaus, Stiftung

## (Selbst‑)Definition der Urologie um 1900

Dass jedoch in Deutschland die Urologie bereits seit Beginn des 20. Jahrhunderts eine Identität besaß, zeigen nicht zuletzt die Etablierung entsprechender Zeitschriften, die Gründung der DGU im Jahr 1906 (erster Kongress 1907) sowie die Beschreibung der Urologie als klinisch und wissenschaftlich relevantes Spezialfach durch Ärzte [48, 49].

Im Lehrbuch von Burckard und Polano 1908 wurde die Urologie folgend definiert:

„Unter Urologie verstehen wir die Lehre von den Erkrankungen der Harnorgane. Die mächtige Förderung, welche Diagnose und Therapie dieser Erkrankungen seit der Einführung der Kystoskopie durch Nitze anfangs der achtziger Jahre des vorigen Jahrhunderts erfahren haben, hatten zur Folge, dass dieses Gebiet mehr und mehr zu einer besonderen Disziplin in der Medizin geworden ist. Wir können nicht verhehlen, daß kaum ein anderes Gebiet so wenig scharf gegenüber der Chirurgie, Gynäkologie und inneren Medizin abzugrenzen ist wie dieses. Von den modernen Spezialvertretern des Faches wird die urologische Klinik sehr weit gefasst, da von ihnen auch die Erkrankungen der männlichen Sexualorgane dazu gerechnet werden“ [50].

Albrecht von Nothafft (1868–1950) [51] wies 1908 auf die zeittypischen Schwierigkeiten in der Abgrenzung des Querschnittsfaches hin:

„Andere Schwierigkeiten ergaben sich bezüglich der Umgrenzung des Begriffes ‚Urologie‘. Für viele ist der Begriff Urologe identisch mit Androloge und Venerologie. Urologen sind aber auch die chirurgischen Spezialisten für Operationen an den jenseits von Prostata und Harnblase gelegenen Harnwegen“ [52].

Leopold Casper (1859–1959) hielt 1913 in seiner Rede als Präsident der alten deutschen Gesellschaft für Urologie fest:

„1. Von der Urologie muß der allgemein Praktiker so viel wissen und können, daß er imstande ist, im Notfalle seinen Pflegebefohlen Hilfe zu bringen.

2. Es muß Ärzte geben, die das Fach voll und ganz beherrschen, die in allen, auch den feinsten Untersuchungs- und Behandlungsmethoden sattelfest sind, so daß sie auch schwierigen Fällen gerecht zu werden vermögen.

3. Es muß Einrichtungen geben, die den Ausbau und die Weiterentwicklung des Faches gewährleisten“ [53].

## Diskussionen über die Einheit der Medizin vs. Spezialistentum vor 1924

Aus den Aussagen von Burckard und Polano sowie Nothafft wird deutlich, dass die Urologie in den ersten beiden Dekaden des 20. Jahrhunderts darum bemüht war, sich von anderen medizinischen Fächern abzugrenzen bzw. die Grenzen zu ihnen zu definieren. Casper fordert, dem Fach sowohl im Medizinstudium als auch in der Spezialistenausbildung Raum bzw. Institutionen einzuräumen. Diese Autoren äußerten im Kontext einer Diskussion über die Einheit oder Ausdifferenzierung der Medizin, welche das Ende des 19. und das beginnende 20. Jahrhundert prägte.

Eine Entschließung des 20. Deutsche Ärztetags aus dem Jahr 1892 zeigt die ambivalente Haltung der Ärzteschaft zu dem Thema:

„Über Spezialärzte.

Der 20. Deutsche Ärztetag hat in seiner Sitzung vom 28. Juni d. J. zu Leipzig folgende Thesen, betreffend die Prüfung der Spezialärzte, aufgestellt:

1. In der Entwickelung des Spezialistentums haben sich Auswüchse gebildet, deren Bekämpfung im Interesse des ärztlichen Standes, sowie im Interesse des Publikums liegt.

Das wichtigste Mittel zur Bekämpfung der Übelstände ist Förderung des Standesbewusstseins durch eine stramme Organisation und eine darauf sich gründende Ärzteordnung.

2. Die Einrichtung einer besonderen Prüfung für Spezialärzte ist zu verwerfen.

3. Ein Verbot jeder näheren spezialistischen Bezeichnung liegt weder im ärztlichen Interesse noch in dem des Publikums“ [54].

Diese Zunahme von Spezialisten war vielen „praktischen Ärzten“, die sich als umfassend ausgebildet ansahen, ein Dorn im Auge. Viele sahen in den Spezialärzten unliebsame Konkurrenten. Bereits 1860 beklagte der Militärarzt und Medizinhistoriker Heinrich Rohlfs (1827–1898) eine drohende Zersplitterung der Medizin: „Fett‑, Knorpel- und Nervenärzte werden nächstens ebenso ‚en vogue‘ sein, wie jetzt die Kehlkopf‑, Gebährmutter, Augen- Ohren und Herzkrankheitenärzte“ [55].

Der Internist Ernst von Leyden (1832–1910) drückte es im Jahre 1881 bei der ersten Sitzung des Berliner Vereins für Innere Medizin noch pointierter aus: „Es giebt gegenwärtig kaum noch Aerzte, fast nur Specialisten, oder man ist Specialist und nebenbei noch Arzt“ [56].

Die Spezialärzte verfügten häufiger über einen höheren Wissensstand aufgrund höher spezieller Fallzahlen. Im Fall der Urologie kam auch einem besonderen, teils auch im Ausland bei spezialisierten Instrumentenmachern erworbenen, preishohen Instrumentenbestand eine Rolle zu: Lithotripsiesets, blinde Lithotriptoren (Fa. Civiale oder Genitle, Fa. Weiss), Katheter (Fa. Porges), Glühlampenzystoskop (Fa. Joseph Leiter), Maisonneuvsches Urethrotom (Fa. Civiale). Zudem hatten sich Spezialisten z. T. im Ausland mit hohen Kosten Kenntnisse durch den Besuch spezialisierter Einrichtungen erworben (Wiener Allgemeine Poliklinik mit Spezialabteilungen, Hôpital St. Louis bzw. Hôpital Necker in Paris).

## Bremer Richtlinien von 1924

Die „Spezialistenfrage“ wurde nach längeren innerärztlichen Diskussionen mit der ersten deutschen Facharztordnung auf dem 43. Deutschen Ärztetag 1924 in Bremen beantwortet [3]. Bereits im Jahre 1922 waren in Sachsen Leitsätze regional in Kraft getreten, in Österreich war zu dieser Zeit die Einführung einer Facharztprüfung gescheitert [2].

In den Leitsätzen zur Facharztfrage, die am 1. Juli 1924 in Kraft traten, wurden 14 „Sonderfächer“, darunter auch die Urologie, aufgeführt, für die Fachärzte zugelassen werden sollten:

„– Chirurgie,

– Frauenkrankheiten und Geburtshilfe,

– Orthopädie,

– Augenkrankheiten,

– Hals‑, Nasen- und Ohrenkrankheiten,

– Haut- und Geschlechtskrankheiten,

– Erkrankungen der Harnorgane (Urologie),

– Nerven- und Geisteskrankheiten,

– Röntgen- und Lichtheilkunde,

– Zahn‑, Kiefer- und Mundkrankheiten (dazu Approbation als Zahnarzt nötig),

– Innere Medizin (einschließlich Nervenkrankheiten),

– Magen-Darm- und Stoffwechselkrankheiten,

– Lungenkrankheiten (Erkrankungen der Luftwege),

– Kinderkrankheiten (nur bis zum vollendeten 13. Lebensjahre. Beratung und Behandlung erwachsener Angehöriger ist grundsätzlich verboten!)“ [57].

Die Bremer Richtlinie war eine Standesregelung, da die Ärzteschaft eine gesetzliche Regelung ausdrücklich ablehnte [58].

Nach den Erfahrungen des Ersten Weltkrieges und der Teils sehr erfolgreichen Zusammenarbeit zwischen Zahnärzten und Chirurgen bei der Behandlung kiefer- und gesichtsverletzter Soldaten war der Facharzt für Zahn‑, Kiefer und Mundkrankheiten für doppelapprobierte Personen mit aufgenommen worden [59]. Dies ist insbesondere deshalb bemerkenswert, weil die Zahnmedizin erst seit 1919 mit eigenem Promotionsrecht an den medizinischen Fakultäten in Deutschland vertreten war [60, 61].

Die Ausbildung war nach Approbation und Praktischem Jahr für die Urologie auf 4 Jahre festgelegt [62], die Ausbildungszeit in „Sprechstunden“ und „Polikliniken“ wurde „zur halben Zeit“ anerkannt, eine Ausbildung in der Chirurgie konnte angerechnet werden. Die Angaben bei der Urologie schwanken zwischen „mindestens 3 bei einem Facharzt“ bis „4 Jahre“ [63], wobei die Zeitzeugen chirurgischer Prägung in der Regel 3 Jahre Chirurgie und 1 Jahr in einer rein urologischen Klinik außerhalb der Universität angaben, da die nachgewiesenen Operationen wie z. B. Zirkumzision, Vasektomie, Hydrozelenoperation, Varikozelenoperation, Ureterolithotomie, Prostatatadenomektomien, Blasenteilresektionen, Nephrektomie/Nephropexie, Nierenbeckenplastik, Zystoskopie und Ureterenkathetereinlage bis in die 1970er-Jahre auch in den chirurgischen Kliniken ausgeübt wurden und nur die eher endoskopischen Eingriffe wie TURB und TURP oder die seltenen Zystektomien mit Harnableitungen oder radikalen Prostatektomien oder Blasenfistelverschlüsse in den dafür spezialisierten Kliniken einigermaßen sicher erlernt werden konnten.

Die Ausbildungsorte sollten Universitätskliniken oder größere Krankenanstalten sein, die von einem entsprechenden Facharzt geleitet wurden, wobei die „Ausbildung sich auf alle Gebiete des Faches“ erstrecken musste. Die Ausbildung in eigener niedergelassener Praxis war nicht zulässig [64].

**Abb. 2:**
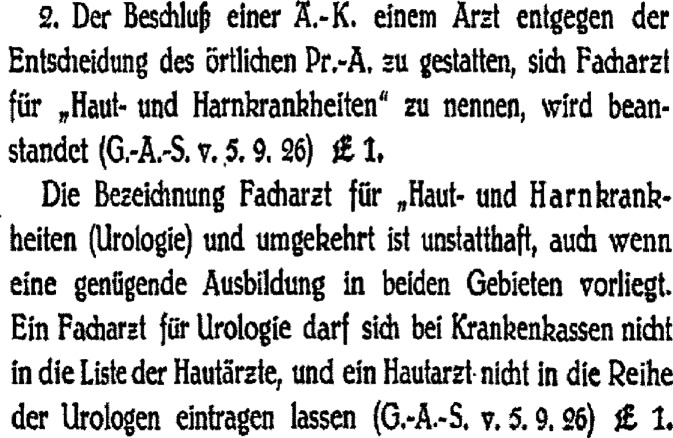
Stuelp 1929 Leitsätze zur Facharztfrage Buchhandlung des Verbandes der Ärzte Deutschland (Hartmannbund), o. O. S 23

**Abb. 3:**
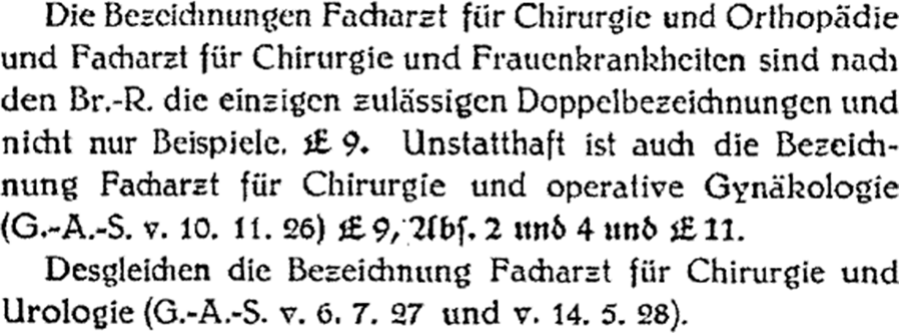
Stuelp 1929 Leitsätze zur Facharztfrage Buchhandlung des Verbandes der Ärzte Deutschland (Hartmannbund), o. O. S 29. (Nach dem Zweiten Weltkrieg setze sich zur Führung mehrerer Facharztbezeichnungen eine andere Regelung durch [67])

Die vor 1924 häufig zu findende Bezeichnung des Spezialarztes für Haut- und Harnkrankheiten, welche die traditionelle Verbindung der Urologie zur Venerologie aufzeigt, wurde in der Folge als nicht mehr zulässig erachtet (vgl. Abb. 2). Auch die Bezeichnung als Facharzt für Chirurgie und Urologie sollte nicht geführt werden (vgl. Abb. 3; [65]). Dies bestätigte etwa noch 1932 Lustig aus der Sicht der Berufspolitik: „Unzulässig ist auch die Bezeichnung ‚Facharzt für innere Medizin und Kinderkrankheiten‘, ‚für Chirurgie und Urologie‘, auch wenn die Ausbildung genügend ist“ [66]. Dies sollte sich erst nach dem Zweiten Weltkrieg ändern.

In den Bremer Leitsätzen wurde 1924 auch festgestellt, dass eine „besondere Prüfung für Fachärzte weder erwünscht noch nötig“ sei. Die Anerkennung als Facharzt erfolgte durch einen Prüfungsausschuss, der den Antrag auf Anerkennung anhand der eingereichten Ausbildungsnachweise zu bescheiden hatte. Eine gesetzliche Regelung wurde abgelehnt, dieser Bereich war „lediglich eine ärztliche Standesfrage, die die praktischen Aerzte und die Fachaerzte in gleicher Weise berührt und nur durch kollegiales Verständnis und durch Zusammenarbeiten beider gelöst werden kann“. Die zuständige „ärztliche Standesorganisation“, die lokale Ärztekammer, erkannte dann eine „fachärztliche“ Tätigkeit als erfolgreich und damit die Führung der entsprechenden Facharztbezeichnung an [68].

Die chirurgischen Ordinarien an den deutschen Hochschulen hatten darüber hinaus vereinbart, dass ein Facharzt für Urologie nur zusammen mit einem Facharzt für Chirurgie zu erteilen wäre, was somit die Ausbildung der ersten universitären Urologengeneration deutlich behinderte, da nur diese Kombination adäquat für eine Berufung auf ein Ordinariat oder eine Chefarztposition wäre.

Schon 1932 stellte hierzu der Chirurg Pels-Leudsden (1866–1944), Greifswald, integralistisch und für die Chirurgie, die für Urologen zu dieser Zeit bei den wenigen rein urologischen Ausbildungskliniken die einzige Möglichkeit war, einen Facharztstatus zu erwerben, geschickt argumentierend fest:

„Ein Chirurg, der nicht zystoskopieren kann, ist meiner Meinung nach kein Vollchirurg und vor allen Dingen nicht ein Facharzt, weil er ja in einem kleineren Ort nicht auch noch einen Urologen neben sich haben wird. Er kann ein ausgezeichneter Chirurg werden und eine große chirurgische Station ohne Urologie betreuen, aber ein Facharzt für Chirurgie ist er nicht“ [69].

Diese Position scheint auch in der Rede als Kongresspräsident der Deutschen Gesellschaft für Chirurgie von Friedrich Voelcker (1872–1955), Halle, im Jahr 1932 durch, der sich vehement gegen „Bindestrich-Chirurgen“ aussprach und dessen Zitat bis in die 1970er-Jahre gerne chirurgischerseits abgedruckt und damit perpetuiert wurde, um die integralistische Fachauffassung weiter zu untermauern und als für die Chirurgie fachkonstituierend anzusehen [70, 71]. Voelker sagte:

„Ich glaube an eine aufsteigende Entwicklung, wenn man dem natürlichen Spiel der Kräfte freie Bahn gibt, ich glaube aber ebenso fest daran, daß man die Entwicklung der Chirurgie drosselt, wenn man sie durch künstliche und unnötige Anerkennung neuer Spezialfächer zerstückelt“ [72].

Zu dieser Zeit behandelten chirurgische Kliniken noch regelhaft urologische Patienten. Besonders vertreten waren in der Allgemeinchirurgie die „offene“ Steinchirurgie an Niere und Harnleiter, Nephrektomien, offene Prostatadenomekotmien und Blasenteilresektionen wie auch Hydrozelenoperationen, Zirkumzisionen und Vasektomien allein unter Epididymitis prophylaktischer Indikation und die Skopie sowie „Blauprobe“ zur groben Einschätzung der Nierenfunktion. Nur die Arbeit in chirurgischen Kliniken lieferten damit die entsprechenden Fallzahlen, die bei den jeweiligen Kammern in den Zeugnissen eingereicht werden mussten.

Zum 1. April 1936 wurde die zentralistische Reichsärztekammer gegründet, welche dann im Jahre 1937 eine neue Berufs- und Facharztordnung erließ. Diese enthielt – lediglich etwas detaillierter – im Wesentlichen die Vorschriften des Ärztevereinsbundes aus der Bremer Richtlinie des Jahres 1924 [70, 73].

## Facharzt für Urologie in der alten Bundesrepublik und DDR

Während in der Bundesrepublik nach dem Zweiten Weltkrieg die Zuständigkeiten der Facharztordnung wieder auf Landesärztekammern dezentralisiert und de facto die Regelungen der Weimarer Republik und der NS-Zeit übernommen wurden, gab die DDR 1956 eine „Anordnung über die Ausbildung und staatliche Anerkennung der Fachärzte“ [74] heraus [75]. Zum Nachweis der Qualifikation war ein Kolloquium abzulegen, welches aus einem theoretischen und einem praktischen Teil bestand. Hierbei wurde von vielen Zeitzeugen eine unterschwellige Prüfung ihrer politischen Haltung geschildert. Ein führender Urologe der DDR, dessen Memoiren früh nach der Wende erschienen, verneinte jedoch, dass es eine solche Gesinnungsprüfung gegeben habe [76].

In der Bundesrepublik wurde im Jahre 1968 die erste Musterweiterbildungsordnung verabschiedet, welche noch deutlicher als vorher den Charakters einer Bildungsordnung hatte [70]. Im gleichen Jahr stellte der erste westdeutsche Ordinarius für Urologie C. E. Alken im Rahmen einer Diskussion um eine neue Approbationsordnung fest, die schließlich 1970 beschlossen und ab 1972 in Kraft treten sollte [77]:

„Die neue Bestallungsordnung sieht seit 1968 folgenden Passus vor: ‚Der Student der Medizin muss in seiner Ausbildung mit den Erkrankungen des Urogenitalsystems vertraut gemacht werden.‘ Damit wird die Lehre urologischer Krankheitsbilder in einem Umfang wie es für den zukünftigen Arzt erforderlich ist, vom Gesetzgeber zur Pflicht gemacht. Es liegt dann nicht mehr im Ermessen einer Fakultät oder eines Chirurgischen Ordinarius, ob Urologie gelesen wird oder nicht“ [78].

Erst ab dem Jahre 1974 wurde dann in Folge eines Urteils des Bundesverfassungsgerichts vom 9. Mai 1972 (Facharzturteil; [79]) eine mündliche Abschlussprüfung bei den Landesärztekammern vor der Verleihung der Gebietsbezeichnung sowie die Pflicht zum Wechsel der Weiterbildungsstätte während der Weiterbildung beschlossen [70]. Weiterhin wurde die Zeit in einer urologischen Klinik und die Rotation von einem Jahr in eine chirurgische Klinik normiert.

Als traditionell chirurgisch geprägtes Fach stieg der Anteil von Frauen in der Urologie in den Nachkriegsjahrzehnten zunächst nur langsam: Beim Urologenkongress 1974 in München stellte Wolfgang Knipper (1920–2005) zum Status des Faches fest: „Unter der Gesamtzahl von 1.073 Urologen haben wir 9 Kolleginnen, die qualifizierte Fachärztinnen für Urologie sind“ [80]. Zum Ende des Jahres 2023 waren 22 % der klinisch tätigen Urologinnen und Urologen Frauen (1479 von 6624), bei den unter 50-Jährigen erreicht der Wert bereits 32 % (973 von 3007; [81–83]).

Ein Urteil des Bundesverfassungsgerichts im Jahre 2000 legte fest, dass Facharztbezeichnungen, die in der Bundesrepublik nicht existent waren, aus der ehemaligen DDR weitergeführt werden dürfen [84, 85]. Dies erklärt, warum die Ärztestatistik zum Ende des Jahres 2023 immer noch 2 Fachärzte für Medizingeschichte ausweist.

## Zusammenfassung und Fazit für die Praxis

Zunächst entwickelten sich die operativen Fächer außerhalb der akademischen Medizin. Ab dem 19. Jahrhundert wurden sie Teil davon, [86] und ab der Wende zum 20. Jahrhundert bildete sich unter akademisch ausgebildeten Ärzten eine Gruppe heraus, die sich selbst als Urologen verstand und die Grenzen des eigenen Faches zu Nachbardisziplinen auslotete. Die Gründung von Fachzeitschriften, Kliniken und Fachgesellschaften kann dabei als Gradmesser der Fachentwicklung gelten. Die Einrichtung von (ordentlichen) Professuren erfolgte in Deutschland erst später.

Mit den Bremer Richtlinien von 1924 gehört der Facharzt für Urologie zu den ersten in Deutschland eingeführten. Gleichzeitig waren bis dahin übliche Doppelbezeichnungen wie Spezialarzt für Haut- und Harnerkrankungen oder für Chirurgie und Urologie nicht mehr statthaft. Der Einfluss der Chirurgie auf die urologische Ausbildung sollte bis in die Nachkriegszeit stark bleiben.

Eine mündliche Facharztprüfung mit theoretischer Wissensabfrage und formalem Leistungsnachweis durch einen „Operationskatalog“ wurde in der alten Bundesrepublik erst ab dem Jahre 1974 eingeführt. Dies wiederum hatte auch internationale Strahlkraft, da angehende Urologen aus dem Ausland nach einer abgeleisteten Assistenzarztzeit in Ost- und Westdeutschland den Facharzt erwerben konnten, der auch im Ausland als qualitativ hochwertige Ausbildung anerkannt ist [87].

Wie in vielen Teilbereichen der Medizin steigt auch in der Urologie der Anteil der Fachärztinnen und erreicht bei den unter 50-Jährigen aktuell ein Drittel.

## Footnotes

1 Das Narrativ, dass C. E. Alken die erste o. Professur für Urologie in Deutschland erhalten habe, ist nur aus dem Narrativ des kalten Krieges zu erklären, erstens gehörte das Saarland bis zum 1. Januar 1957 zu Frankreich als Staat, „der in einer Art wirtschaftlichem Protektoratsverhältnis zu Frankreich“ als „autonomes, demokratisches und sozial geordnetes, wirtschaftlich an Frankreich angeschlossenes Land“ steht. Alken selber rekurriert immer auf ein Extraordinariat 1948 Universität Nancy. Weiterhin hatte Martin Stolze seine o. Professur ebenfalls am 1.10.1958 erhalten und war vorher bereits ao. Professor seit 1955 in Halle ((UAHW, Rep. 11, PA 37481, Stolze Martin)) wie Alken, der jedoch an eine französische Universität akademisch angeschlossen war. In den eigenen Erinnerungen von Medizinern wird gerne a o und o Professur und die Bestellung zum Chefarzt einer universitären Krankenhausabteilung rückblickend nicht mehr weiter differenziert, wobei es sich um unterschiedliche Schritte im universitären Verwaltungskontext handelt. Weiterhin wurde die Saar aus deutscher Sicht als deutsch auch nach dem zweiten Weltkrieg wahrgenommen.
